# Associations Between High Plasma Methylxanthine Levels, Sleep Disorders and Polygenic Risk Scores of Caffeine Consumption or Sleep Duration in a Swiss Psychiatric Cohort

**DOI:** 10.3389/fpsyt.2021.756403

**Published:** 2021-12-20

**Authors:** Nermine Laaboub, Mehdi Gholam, Guibet Sibailly, Jennifer Sjaarda, Aurélie Delacrétaz, Céline Dubath, Claire Grosu, Marianna Piras, Nicolas Ansermot, Severine Crettol, Frederik Vandenberghe, Carole Grandjean, Franziska Gamma, Murielle Bochud, Armin von Gunten, Kerstin Jessica Plessen, Philippe Conus, Chin B. Eap

**Affiliations:** ^1^Unit of Pharmacogenetics and Clinical Psychopharmacology, Department of Psychiatry, Centre for Psychiatric Neuroscience, Lausanne University Hospital, University of Lausanne, Prilly, Switzerland; ^2^Center of Psychiatric Epidemiology and Psychopathology, Department of Psychiatry, Lausanne University Hospital, University of Lausanne, Prilly, Switzerland; ^3^Les Toises Psychiatry and Psychotherapy Center, Lausanne, Switzerland; ^4^Centre for Primary Care and Public Health (Unisanté), University of Lausanne, Lausanne, Switzerland; ^5^Service of Old Age Psychiatry, Department of Psychiatry, Lausanne University Hospital, University of Lausanne, Prilly, Switzerland; ^6^Service of Child and Adolescent Psychiatry, Department of Psychiatry, Lausanne University Hospital, University of Lausanne, Prilly, Switzerland; ^7^Service of General Psychiatry, Department of Psychiatry, Lausanne University Hospital, University of Lausanne, Prilly, Switzerland; ^8^School of Pharmaceutical Sciences, University of Geneva, University of Lausanne, Geneva, Switzerland; ^9^Center for Research and Innovation in Clinical Pharmaceutical Sciences, University of Lausanne, Lausanne, Switzerland; ^10^Institute of Pharmaceutical Sciences of Western Switzerland, University of Geneva, University of Lausanne, Geneva, Switzerland

**Keywords:** sleep disorders, psychiatry, plasma caffeine level, plasma methylxanthines, polygenic risk score (PRS)

## Abstract

**Objective:** We first sought to examine the relationship between plasma levels of methylxanthines (caffeine and its metabolites) and sleep disorders, and secondarily between polygenic risk scores (PRS) of caffeine consumption or sleep duration with methylxanthine plasma levels and/or sleep disorders in a psychiatric cohort.

**Methods:** Plasma levels of methylxanthines were quantified by ultra-high performance liquid chromatography/tandem mass spectrometry. In inpatients, sleep disorder diagnosis was defined using ICD-10 “F51.0,” sedative drug intake before bedtime, or hospital discharge letters, while a subgroup of sedative drugs was used for outpatients. The PRS of coffee consumption and sleep duration were constructed using publicly available GWAS results from the UKBiobank.

**Results:** 1,747 observations (1,060 patients) were included (50.3% of observations with sleep disorders). Multivariate analyses adjusted for age, sex, body mass index, setting of care and psychiatric diagnoses showed that patients in the highest decile of plasma levels of methylxanthines had more than double the risk for sleep disorders compared to the lowest decile (OR = 2.13, *p* = 0.004). PRS of caffeine consumption was associated with plasma levels of caffeine, paraxanthine, theophylline and with their sum (β = 0.1; 0.11; 0.09; and 0.1, p_corrected_ = 0.01; 0.02; 0.02; and 0.01, respectively) but not with sleep disorders. A trend was found between the PRS of sleep duration and paraxanthine levels (β = 0.13, p_corrected_ = 0.09).

**Discussion:** Very high caffeine consumption is associated with sleep disorders in psychiatric in- and outpatients. Future prospective studies should aim to determine the benefit of reducing caffeine consumption in high caffeine-consuming patients suffering from sleep disorders.

## Introduction

Sleep disorders are a worldwide public health problem and their prevalence rates can vary considerably (1). There are several types of sleep disorders (2) and, although efforts have been made to better homogenize their definition, to date, sleep disorders remain poorly described (3). Interestingly, many genes have been associated with sleep regulation, some of them being associated with specific types of sleep disorders (4). However, only a few genes are known to be responsible for severe sleep disorders (5). Eighty to 90% of major depressive patients suffer from insomnia (6–8), which appears before (>40%) or at the same time (>22%) as mood disorder symptoms (9). Thus, sleep and psychiatric disorders share a complex two-way relationship.

The consequences of sleep disorders can be minor such as fatigue or sleepiness, but can sometimes lead to more serious disturbances, including obesity (10), diabetes (11), high blood pressure (12), metabolic syndrome (13), and cardiovascular diseases (14), which can increase mortality risk (15). Severe insomnia is associated with higher frequency of hypertriglyceridemia and metabolic syndrome in major depressive patients (16, 17).

Epidemiological studies in psychiatric cohorts reported that elderly patients as well as women are more likely to suffer from sleep disorders (18, 19). Psychotropic drugs such as antidepressants, antipsychotics and mood stabilizers can also disrupt sleep (20). In addition, caffeine, the most consumed psychoactive substance in the world, can affect sleep and promote wakefulness by antagonizing adenosine A_1_ and A_2_ brain and heart receptors (21). Therefore, caffeine intake late in the day may contribute to poor sleep or exacerbate preexisting sleep problems (22, 23). Caffeine (1,3,7-trimethylxanthine) is mainly metabolized by cytochrome P450 1A2 (CYP1A2) to paraxanthine, theophylline and theobromine (24, 25). Thus, it is expected that high plasma caffeine levels with low metabolite levels would be measured shortly after caffeine intake, while the reverse would be found over time. Genome-wide association studies (GWAS) have identified eight independent loci associated with caffeine consumption at the genome-wide significance level and the most strongly associated loci are on or near *CYP1A2, CYP1A1* and *aryl-hydrocarbon receptor* (*AHR*) genes, the latter affecting CYP1A activity (26, 27).

In psychiatric patients, higher caffeine consumption has been reported compared to the general population (28–30), with high caffeine consumption being associated with metabolic disturbances (31). Thus, sleep disorders and/or high caffeine consumption can both contribute to the high prevalence of metabolic disorders observed in the psychiatric population (32).

With the exception of our own study (31), all the above-mentioned studies in psychiatric patients used self-reported caffeine intake as a proxy for caffeine exposure, which may be prone to uncertainties. The main objective of the present study was therefore to examine the relationship between plasma levels of methylxanthines (caffeine and its metabolites) and the occurrence of sleep disorders in a psychiatric cohort which has, to our knowledge, never been investigated. In addition, we sought to assess whether polygenic risk scores (PRS), a summarized measure of an individual's genetic risk across the entire genome, integrating genes previously linked by GWAS studies to caffeine intake (33) or sleep duration (34), are associated with plasma levels of methylxanthines and/or with sleep disorders in our psychiatric cohort.

## Methods

### Study Design

Since 2007, a longitudinal observational study (PsyMetab) has been ongoing at the Department of Psychiatry of the Lausanne University Hospital as described previously (35). Psychiatric patients at the university hospital (in- and outpatients) and at a private center starting or already receiving a psychotropic treatment known to have a potential risk of inducing metabolic disturbances (list in Supplementary Table 1) were included. Monitoring of clinical parameters (e.g., weight, waist circumference) was conducted and blood samples were taken at the start of psychotropic treatment and at 1, 3, 12 months and once a year to monitor metabolic parameters (e.g., lipids). Additional analyses (e.g., methylxanthine assays) were performed on samples from patients who gave written informed consent.

### Quantification of Methylxanthine Plasma Levels

Plasma levels of caffeine, paraxanthine, theophylline and theobromine were quantified by ultra-high performance liquid chromatography (Waters ACQUITY UPLC system) coupled to a tandem quadrupole mass spectrometer (Waters TQD with electrospray ionization or Waters Xevo TQ-S with UniSpray ion source). The method was validated according to international guidelines using a stable isotope-labeled internal standard for each analyte (detailed method available on request). Limit of quantification for all analytes was 5 ng/ml. Plasma samples were collected between December 2007 and August 2018. In order to estimate more precisely the exposure to caffeine and metabolites, and to reduce the heterogeneity due to various time intervals between caffeine intake and blood collections, the sum of caffeine, paraxanthine and theophylline was used in the analyses.

### Sleep Disorders

Sleep disorders were defined by the presence of at least one of the following criteria during the clinical follow-up when plasma methylxanthine levels were measured. For inpatients, the ICD-10 “F51.0” diagnosis (non-organic insomnia), if available in medical files or the prescription of sedative drugs before bedtime, taking into account the dosage and the prescription condition (for insomnia) (Supplementary Table 2), was used. When these two variables were not available, the hospital discharge letter of the corresponding hospital stay was screened to detect indications of sleep disorders including difficulty in initiating or maintaining sleep, insomnia or repeated nocturnal awakenings. For outpatients, those receiving the following sedative drugs, melatonin, zolpidem, zopiclone, and herbal sedatives (valerian and hops) were considered to be with sleep disorders and those not receiving such drugs were considered to be without sleep disorders. Outpatients receiving the other sedative drugs listed in Supplementary Table 2 were excluded as information on the time of drug intake and dosage, as well as clinical reports, were not available in the electronic files.

### Construction of Polygenic Risk Scores (PRS)

All participants were genotyped using the Global Screening Array (GSA) v2 from Illumina with the multiple disease option (MD) chip and standard quality control filters applied. Data was imputed on the Michigan Imputation Server and SNPs with INFO score > 0.3 were retained. The PRS were then constructed in the PsyMetab cohort for both caffeine consumption and sleep duration using beta estimates from publicly available GWAS computed and made available by the Neale Lab, which has conducted GWAS across thousands of traits within the UKBiobank (UKB), a large population-based cohort (33). Specifically, the caffeine consumption GWAS (Data Field 100240) and the PRSice software (36) were used to derive the PRS of caffeine consumption within PsyMetab using a *p* < 0.10 threshold for SNP inclusion and the default clumping parameter (clump-r2 0.10), resulting in 34,537 SNPs in the PRS. Finally, this PRS was scaled to have a mean of 0 and SD of 1.

A PRS with 91 SNPs associated with sleep duration was derived from the results of the UKBiobank GWAS study (34). Two SNPS (rs2139261, rs142180737) could not be included in this PRS since neither these SNPs nor their proxies were available in our GWAS results.

PRS was derived in Caucasian individuals only in PsyMetab, as determined by the SNPweights software (37). The two PRSs were tested for associations with plasma caffeine and/or its metabolite levels [log caffeine, log paraxanthine, log theophylline, log theobromine, log (caffeine + paraxanthine + theophylline)] and with sleep disorders using linear regression. Models were adjusted for sex, age, age^2^, BMI, setting of care (inpatient/outpatient), psychiatric diagnosis and the first 20 principal components (PCs).

### Other Covariates

The following variables were included in the analyses as they are related to sleep disturbance and/or plasma methylxanthine levels: age, sex, smoking habits and daily activity (dichotomized as smoker or non-smoker and engaging in physical activity for <30 min, between 30 and 60 min, or more than 60 minutes per day, respectively), body mass index (BMI; calculated by dividing weight in kilograms by height in square meters), psychiatric diagnosis (defined according to ICD-10), blood pressure and plasma creatinine levels.

### Statistical Analyses

Because of the longitudinal study design, some patients had multiple observations (average time between observations: 12.4 months) and the sleep disorder status may vary over time. All observations were therefore included in the analyses, with two groups being defined as with and without sleep disorders. Descriptive data are shown as numbers and percentages for categorical variables or median and interquartile range (IQR) for continuous variables. In order to compare sleep disorder groups, Wilcoxon-Mann-Whitney rank-sum tests or the Chi-squared test were used, depending on the variable type. For multivariate analyses, generalized linear mixed-effects models were used to examine the association between plasma levels of methylxanthines (log transformed) and sleep disorders in the whole cohort.

As the definition of sleep disorder varies considerably according to the setting of care, stratified analyses were conducted using the same univariate and multivariate models described above in inpatients and outpatients separately. Specifically in inpatients, robust linear regression was applied using the first plasma methylxanthine measurement per patient in order to investigate the association between length of stay and plasma levels of methylxanthines. To determine whether plasma methylxanthine levels were higher in our psychiatric population compared to the general population, a general population cohort from the Swiss Kidney Project on Genes in Hypertension “SKIPOGH” study, which is a family- and population-based study exploring genetic and environmental determinants of blood pressure, was used (Supplementary Figure 1) (38). Participants were recruited from November 2009 to April 2013 (SKIPOGH1) and from March 2013 to December 2016 (SKIPOGH2) in the Swiss cities of Lausanne, Geneva and Bern and caffeine consumption was assessed by plasma methylxanthine levels. Analyses were conducted using PsyMetab outpatients only in order to reduce heterogeneity and to ensure comparability between the two cohorts.

As the missing data were completely at random and 1,341 (76.7%) observations had no missing data (Supplementary Figure 2), missing values were replaced using 50 multivariate imputation by chained equations (Mice) Package in R (39, 40). Data preparation and univariate analyses were conducted using Stata 16.0 (StataCorp; College Station, Texas), and multivariate analyses were performed using the R environment for statistical computing version 3.6.1. *P*-values ≤ 0.05 were accepted as statistically significant for all analyses except those with PRS (see Results).

## Results

### Methylxanthine Levels and Sleep Disorders

#### Whole Sample

**Figure 1:**
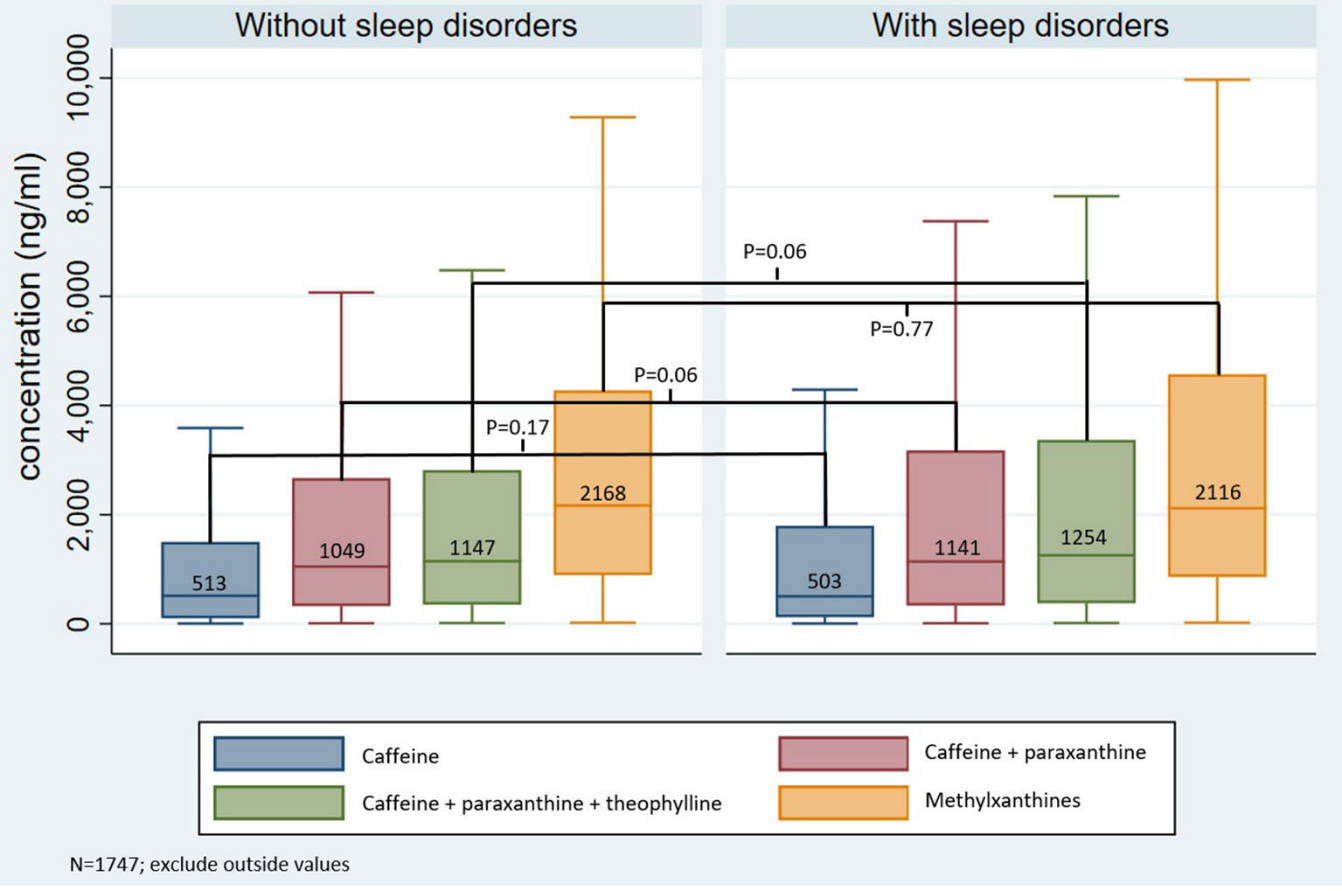
Plasma methylxanthine levels in groups with and without sleep disorders. ng, nanogram; ml, milliliter, Methylxanthines: caffeine + paraxanthine + theophylline + theobromine.

1,747 observations (from 1,060 patients) were included in the descriptive and multivariate analyses (Supplementary Figure 3). Sleep disorders were noted for 880 observations (50.3% of the cohort). The median age and percentages of sedentary patients, females and inpatients were higher in the group with sleep disorders (median age 43 vs. 35 years old, *p* < 10^–4^; 51% doing <30 min of activity daily vs. 35%, *p* < 10^–3^; 53 vs. 47%, *p* = 0.02; 79 vs. 25%, *p* < 10^–3^, respectively, Table 1). No significant differences were observed in BMI, smoking status, psychiatric diagnoses, blood pressure and plasma creatinine levels between the two groups (Table 1). Caffeine and total methylxanthine plasma levels were identical in the two groups with and without sleep disorders (503 vs. 513 ng/ml, *p* = 0.17; 2,116 vs. 2,168 ng/ml, *p* = 0.77, respectively), whereas a trend was observed for higher caffeine + paraxanthine (1,141 vs. 1,049 ng/ml, *p* = 0.06) and caffeine + paraxanthine + theophylline (1,254 vs. 1,147 ng/ml, *p* = 0.06) levels in patients with sleep disorders (Figure 1).

**Table 1:** Clinical and demographic data comparing the groups with and without sleep disorders.

	**Sleep disorders NO (*N* = 867)**	**Sleep disorders YES (*N* = 880)**	***P*-value**
Age (median, IQR) years	**35 (27–50)**	**43 (31–60)**	**<10** ^**−4**^
Sex (Female) (*N*, %)	**407 (47)**	**464 (53)**	**0.02**
BMI (median, IQR) kg/m^2^	24.6 (21.7–28.7)	24.7 (21.9–28.7)	0.95
Smokers (*N*, %)			0.06
Yes	435 (52)	383 (48)	
No	395 (48)	418 (52)	
Psychiatric diagnosis (*N*, %)^a^			0.87
Other disorders	148 (17)	149 (17)	
Psychotic disorders	302 (35)	309 (35)	
Schizoaffective disorders	124 (14)	116 (13)	
Bipolar disorders	122 (14)	138 (16)	
Depression	171 (20)	168 (19)	
Daily activity (*N*, %)			**<10^−3^**
<30 min/day	**274 (35)**	**346 (51)**	
30-60 min/day	257 (33)	213 (31)	
>60 min/day	250 (32)	123 (18)	
Setting of care (*N*, %)			**<10^−3^**
Outpatients	646 (75)	188 (21)	
Inpatients	**221 (25)**	**692 (79)**	
Blood Pressure (median, IQR) mmHg			
Systolic	120 (110-131)	123 (111-133)	0.13
Diastolic	80 (70-86)	79 (70-86)	0.65
Creatinine (median, IQR) μmol/l	76 (66-87)	75 (66-85)	0.45
Length of stay^*^ (median, IQR) days	56 (32-105)	63 (33-109)	0.32

*Bold values indicate significant results*.

*IQR, Interquartile range; N, Number; BMI, Body Mass Index; kg, kilogram; m^2^, square meter; min, minutes; mmHg, millimeter of mercury; μmol, micromole; l, liter*.

a *Psychiatric diagnoses were based on ICD-10 classification, and were classified as: Other disorders [F00-F19; F34-F99] | Psychotic disorders [F20-F24; F28-F29] | Schizoaffective disorders [F25] | Bipolar disorders [F30-F31] | Depression [F32-F33]*.

*N varies between variables due to missing values (see Methods)*.

* *Only for inpatients*.

When compared to outpatients, inpatients were older (median age 46 vs. 40 years, *p* < 10^−**4**^), predominantly female (53 vs. 46%, *p* = 0.003), had lower BMI (median BMI 25 vs. 26.4 kg/m^2^, *p* < 10^−**4**^), were less often smokers (47 vs. 53%, *p* = 0.02), and were more sedentary (doing <60 min of activity daily 85 vs. 65%, *p* < 10^−3^) (Supplementary Table 3). No significant differences were observed in psychiatric diagnoses, systolic blood pressure and plasma creatinine levels between the two groups. Higher plasma levels of caffeine and all of its metabolites were measured in outpatients as compared to inpatients (Supplementary Figure 4).

Multivariate analysis showed that the risk of sleep disorders increased by 17% when age increased by 10 years (*p* < 10^−3^), by 3% when BMI increased by one unit (*p* = 0.01), and by 8% when log caffeine and its two metabolites increased by one unit (*p* = 0.04, Table 2). Inpatients were 11 times more at risk of suffering from sleep disorders than outpatients (*p* < 10^−3^). No significant association was observed between sleep disorders and sex, smoking status and psychiatric diagnoses.

**Table 2:** Association between sleep disorders and clinical variables (whole cohort, *N* = 1,747).

	**Odds Ratio**	**95% CI**	***P*-value**
Age (10 Years)	**1.17**	1.09−1.26	**<10** ^**−3**^
Sex (female)	1.03	0.82−1.3	0.78
BMI (kg/m^2^)	**1.03**	1.00−1.05	**0.01**
Smokers (Yes)	1.05	0.82−1.34	0.69
Setting of care (inpatients)	**11.13**	8.8−14.09	**<10** ^**−3**^
Log (caffeine + paraxanthine + theophylline)	**1.08**	1.00−1.16	**0.04**
Psychotic disorders^*^	1.09	0.76−1.56	0.63
Schizoaffective disorders^*^	1.02	0.66−1.56	0.93
Bipolar disorders^*^	1.21	0.8−1.82	0.36
Depression^*^	1.02	0.7−1.5	0.90

*Bold values indicate significant results. CI, confidence interval; BMI, Body Mass Index; kg, kilogram; m^2^, square meter; Log, logarithm*.

*Psychiatric diagnoses were based on ICD-10 classification and were classified as: Other disorders [F00-F19; F34-F99] | Psychotic disorders [F20-F24; F28-F29] | Schizoaffective disorders [F25] | Bipolar disorders [F30-F31] | Depression [F32-F33]*.

* *All diagnoses were compared to “other disorders.”*

After dividing plasma levels of caffeine and its two metabolites into six quantiles [(Q1 ≤ 10%), (10% < Q2 ≤ 25%), (25% < Q3 ≤ 50%), (50% < Q4 ≤ 75%), (75% < Q5 ≤ 90%), and (Q6 > 90%)], the highest decile (Q6) of caffeine and its two-metabolite concentration had more than double the risk of suffering from sleep disorders when compared to the lowest one (Q1) (OR = 2.13, *p* = 0.004) (Table 3). Q6 of caffeine concentration was also up to two times more likely to suffer from sleep disorders compared to Q1 when analyzing plasma levels of caffeine only, caffeine + paraxanthine or caffeine plus its three metabolites (OR = 2.01, *p* = 0.008; OR = 2.05, *p* = 0.006; OR = 1.68, *p* = 0.045, respectively; data not shown).

**Table 3:** Association between sleep disorders and clinical variables (whole cohort; quantile of plasma caffeine + paraxanthine + theophylline; (*N* = 1,747).

	**Odds ratio**	**95% CI**	***P*-value**
Age (10 Years)	**1.17**	1.09−1.25	**<10** ^**−3**^
Sex (Female)	1.03	0.81−1.29	0.83
BMI (kg/m^2^)	**1.03**	1.00−1.05	**0.01**
Smokers (Yes)	1.02	0.8−1.31	0.84
Setting of care (inpatients)	**11.25**	8.88−14.26	**<10** ^**−3**^
Q6 vs. Q1^*^	**2.13**	1.27−3.57	**0.004**
Q5 vs. Q1^*^	1.29	0.8−2.06	0.29
Q4 vs. Q1^*^	1.18	0.77−1.81	0.44
Q3 vs. Q1^*^	1.17	0.77−1.78	0.46
Q2 vs. Q1^*^	1.24	0.79−1.96	0.34
Psychotic disorders^§^	1.09	0.76−1.55	0.64
Schizoaffective disorders^§^	1.01	0.66−1.55	0.95
Bipolar disorders^§^	1.22	0.81−1.83	0.35
Depression^§^	1.03	0.7−1.51	0.87

*Bold values indicate significant results. CI, confidence interval; BMI, Body Mass Index; kg, kilogram; m^2^, square meter; Log, logarithm*.

* *Log (caffeine + paraxanthine + theophylline) (ng/ml): Q1 ≤ 4.57; 4.57 < Q2 ≤ 5.9; 5.9 < Q3 ≤ 7.08; 7.98 < Q4 ≤ 8.05; 8.05 < Q5 ≤ 8.68; Q6 > 8.68*.

*Psychiatric diagnoses were based on ICD-10 classification, and were classified as: Other disorders [F00-F19; F34-F99] | Psychotic disorders [F20-F24; F28-F29] | Schizoaffective disorders [F25] | Bipolar disorders [F30-F31] | Depression [F32-F33]*.

§ *All diagnoses were compared to “other disorders.”*

#### Inpatients

In inpatients (*N* = 913, Supplementary Table 4), age, BMI and psychotic, schizoaffective, bipolar and depressive disorders (when compared to other disorders) were significantly associated with the occurrence of sleep disorders. A trend was observed for a higher frequency of sleep disorders in Q6 of caffeine plus paraxanthine and theophylline concentration (OR= 2.14; *p* = 0.056), whereas Q6 of caffeine alone was 3 times (OR = 3.12; *p* = 0.008; Supplementary Table 5) more likely to suffer from sleep disorders compared to Q1 (for caffeine and paraxanthine: OR = 2.63, *p* = 0.02; data not shown).

No association was observed between plasma levels of caffeine and/or its metabolites with length of stay, while age, psychotic, schizoaffective and bipolar disorders were positively associated with longer length of stay, opposite of females and smokers, who were associated with shorter length of stay (Supplementary Table 6).

#### Outpatients

In contrast to inpatients, outpatients (*N* = 834) suffering from schizoaffective disorders and depression were 65% and 44% less likely to have sleep disorders, respectively (OR = 0.35, *p* = 0.002; and OR = 0.56, *p* = 0.03, respectively, when compared to other disorders; data not shown). When comparing Q6 of caffeine plus paraxanthine plus theophylline with Q1, Q6 was 78% more at risk of having sleep disorders, although this difference did not reach statistical significance (*p* = 0.13; data not shown). However, Q6 of caffeine and its two metabolites was 66% more at risk of suffering from sleep disorders (*p* = 0.005) than all other outpatients (Supplementary Table 7) and Q6 of all methylxanthines were 2.2 times more at risk compared to Q1 (*p* = 0.048; data not shown).

#### Caffeine and Metabolite Plasma Levels in Psychiatric Outpatients and the General Population

Higher caffeine plasma levels were measured in psychiatric outpatients as compared to subjects from general population cohorts (737 vs. 591 ng/ml, *p* = 0.01; 737 vs. 504 ng/ml, compared to SKIPOGH1 and SKIPOGH2, respectively; see Supplementary Tables 8, 9 for more results).

### PRS

#### PRS of Caffeine Consumption

In Caucasian patients (*N* = 669), a positive association was found between the PRS of caffeine consumption built with 34,537 SNPs previously associated with caffeine consumption (33) and log caffeine, log paraxanthine, log theophylline, and log (caffeine + paraxanthine + theophylline) after adjusting for multiple testing (Supplementary Table 10). On the other hand, PRS of caffeine consumption was not associated with sleep disorders (*p* = 0.18).

#### PRS of Short Sleep Duration

After adjusting for multiple testing, a trend was found for positive associations between PRS built on 91 SNPs previously associated with sleep duration (34) with log (caffeine) and log (paraxanthine) (*p* = 0.10 and *p* = 0.09, respectively; Supplementary Table 11). No significant association was found with the occurrence of sleep disorders.

## Discussion

A 50% prevalence rate of sleep disorders was measured in the present psychiatric cohort, which is in agreement with previous epidemiological studies in psychiatry reporting prevalence rates between 30 and 80% depending on the definition used (1, 41). The main result of the present study is that caffeine consumption, estimated by caffeine and its metabolite plasma levels, was associated with the occurrence of sleep disorders in psychiatry, with patients in the highest decile of caffeine and its metabolite plasma levels being more than two times more prone to sleep disorders. This is in agreement with previous population-based studies associating increased caffeine consumption with sleep disorders (42–44). Higher caffeine and its metabolite plasma levels were found in our psychiatric cohort as compared to a population-based cohort, which is in agreement with previous studies (28, 45, 46). Of note, using self-estimated caffeine consumption, one study previously reported that daily caffeine consumers in a psychiatric cohort had shorter sleep duration (46). To our knowledge, the present study is the first to show that high plasma levels of caffeine, paraxanthine and/or theophylline, but not theobromine, are significantly associated with sleep disorders in psychiatric inpatients and outpatients.

Summing up, caffeine, paraxanthine and theophylline can be considered an active moiety of caffeine on adenosine receptors (47). However, theobromine was not considered in that moiety because of its primary provenance being chocolate (48), with higher plasma levels of theobromine measured in outpatients possibly being due to decreased consumption of chocolate while in hospitals.

Patients included in the present study were treated with psychotropic drugs with well-known metabolic side effects (49). In addition to the two- to three-fold increased risks of sleep disorders, psychiatric patients with high caffeine consumption have been previously reported to be two, four, and five times more prone to suffer from non-HDL hypercholesterolemia, overweight and hypertriglyceridemia, respectively (31). Although a causality link cannot be demonstrated in the present study, sleep disorders are also interlinked with metabolic disturbances (13, 14). In psychiatric patients treated with weight gain-inducing drugs, high caffeine consumption may increase the risk of metabolic disturbances as well as sleep disorders, which in turn could induce metabolic disturbances. Such patients should therefore be closely monitored, with prevention and awareness programs set up to counter excessive caffeine consumption.

The present study identified other correlates of sleep disorders, including age, BMI, type of setting of care, and psychiatric diagnosis, in agreement with previously published studies. Older age has thus been found to be strongly associated with sleep disorders (1, 9, 41). High BMI has also been associated with sleep disorders (10). Inpatients were 11 times more likely to suffer from sleep disorders than outpatients, which is in agreement with a previous study (41). However, the effect size may be too high due to the strict definition of outpatient sleep disorders, which probably results in a large number of false negatives. Inpatients with psychotic, schizoaffective, bipolar and depressive disorders were more likely to have sleep disorders when compared to patients with other diagnoses, which is in line with previous studies (6–8, 50, 51). On the other hand, outpatients with schizoaffective disorders or depression are less likely to have sleep disorders, which is most probably explained by the less severe illness in outpatients. Finally, several studies reported that women are more likely to have sleep disorders, both in the general and psychiatric populations (1, 9), but no significant association was found between sex and sleep disorders in the present cohort, which can tentatively be explained by the presence of false negatives due to sleep disorder classification.

Our results confirm the positive correlation between the PRS of caffeine intake and measured caffeine consumption as previously described in several studies, both in the general population and in psychiatric cohorts (27, 52). However, an association between sleep disorders and this PRS could not be demonstrated, which is not surprising since sleep disorders are influenced by multiple factors besides caffeine intake. Regarding PRS of sleep duration, association trends were observed with log (caffeine) and log (paraxanthine) after correction for multiple testing. These results suggest that some genes previously associated with sleep duration, which are used for the present PRS, might actually exert their effect(s) *via* caffeine plasma levels. However, no association was observed between PRS of sleep duration and sleep disorders, probably because of the classification used for sleep disorders in the present study which very partially reflects sleep duration. Secondly, the PRS used was derived from genetic studies in the general population, which can differ from the psychiatric population.

This study has several limitations. First, this is an observational longitudinal study and no causal relationship can be established between caffeine exposure and sleep disorders. However, our results are in line with multiple studies performed in the general population (42, 43, 53) and with the well-known mechanisms of action of caffeine and metabolites on the adenosine receptors (21, 47). Secondly, a sleep disorder diagnosis (ICD-10: F51.0) was available for only one observation. Indeed, psychiatric patients are often treated for major illnesses and eventually admitted to the hospital in the context of a worsening of their disorder(s). Only primary diagnoses are retained; sleep disorders often considered a symptom and therefore scarcely diagnosed. Thus, focusing on medication to define sleep disorders may possibly raise the number of false negatives, especially in outpatients. However, despite our restrictive criteria, we have been able to show an association between plasma methylxanthine levels and the occurrence of sleep disorders. Third, we were not able to better characterize sleep disorders experienced by patients, which should be investigated in future studies.

Despite these limitations, our study has several strengths. It was conducted in inpatients and outpatients and in two different settings (university and ambulatory hospitals and a private center). Furthermore, most studies of sleep disorders focus on a specific diagnosis such as depression. We adjusted our multivariate analysis models across all psychiatric diagnoses, allowing us to study more sleep disorder risk factors, increasing the potential applicability and relevance of the results to a large psychiatric population. The association between caffeine consumption and sleep disorders was investigated using plasma methylxanthine levels, which is probably a more reliable measure than patient-reported consumption. In addition, the determination of metabolite plasma levels in addition to caffeine allows taking into account the variability of caffeine metabolism.

## Conclusion

Among psychiatric patients, high caffeine consumers are more likely to have sleep disorders. Future prospective studies should aim to demonstrate whether reducing caffeine consumption in patients suffering from sleep disorders could improve sleep and/or reduce the prescription of sedative drugs.

## Data Availability Statement

The datasets presented in this article are not readily available because, at the time of publication, the SwissUbase repository wasn't operational. Requests to access the datasets should be directed to the corresponding author.

## Ethics Statement

The studies involving human participants were reviewed and approved by the Ethics committee of Vaud (CER-VD), Switzerland. The patients/participants provided their written informed consent to participate in this study. Written informed consent was not obtained from the individual(s), nor the minor(s)' legal guardian/next of kin, for the publication of any potentially identifiable images or data included in this article.

## Author Contributions

CE had full access to all of the data in the study and takes responsibility for the integrity of the data and the accuracy of the data analysis. Study concept and design was provided by CE. Acquisition of data was provided by NL, CD, ClG, MP, AD, GS, FV, NA, SC, CaG, MB, FG, KP, AG, and PC. Analyses and interpretation were provided by NL, JS, AD, and MG. Drafting of the manuscript was provided by NL. Statistical analysis was provided by NL, JS, and MG. CE, PC, and KP obtained funding for the study. Administrative, technical, or material support was provided by FG, KP, AG, PC, and CE. Critical revision of the manuscript for important intellectual content was provided by all authors. All authors contributed to the article and approved the submitted version.

## Funding

This work has been funded in part by the Swiss National Research Foundation (CE and PC: 320030-120686, 324730-144064, and 320030-173211; CE, PC, and KP: 320030_200602; MB: 33CM30-124087/140331). The funding sources had no role in the writing of the manuscript or in the decision to submit it for publication.

## Conflict of Interest

CE received honoraria for conferences from Janssen-Cilag, Lundbeck, Otsuka, Sandoz, Servier, Sunovion, Vifor-Pharma, and Zeller in the past 3 years. FV received honoraria for conferences or teaching CME courses from Forum fur Medizinische Fortbildung in the past 3 years. The remaining authors declare that the research was conducted in the absence of any commercial or financial relationships that could be construed as a potential conflict of interest.

## Publisher's Note

All claims expressed in this article are solely those of the authors and do not necessarily represent those of their affiliated organizations, or those of the publisher, the editors and the reviewers. Any product that may be evaluated in this article, or claim that may be made by its manufacturer, is not guaranteed or endorsed by the publisher.
