# Supplementary material for: Associations Between High Plasma Methylxanthine Levels, Sleep Disorders and Polygenic Risk Scores of Caffeine Consumption or Sleep Duration in a Swiss Psychiatric Cohort
Source: Front Psychiatry. 2021 Dec 20;12:756403. doi: 10.3389/fpsyt.2021.756403 (PMC8721597; doi:10.3389/fpsyt.2021.756403)
Supplement: Supplementary file 1 [file Data_Sheet_1.docx]

**Supplementary Table 1:** **Psychotropic drugs included in the metabolic follow-up**

| **Antipsychotics** | | **Antidepressants** | | | **Mood Stabilizers** |
| --- | --- | --- | --- | --- | --- |
| **Atypical** | **Typical** | **Tricyclic** | **Others** | |  |
| Amisulpride  Aripiprazole  Asenapine  Brexpiprazole  Cariprazine  Clozapine  Lurasidone  Olanzapine  Paliperidone  Quetiapine  Risperidone  Sertindole | Chlorprothixene  Flupentixol Haloperidol  Levomepromazine  Pipamperone  Promazine  Sulpiride  Tiapride  Zuclopenthixol | Amitriptyline  Clomipramine  Doxepine Nortriptyline Opipramol Trimipramine | | Mirtazapine | Carbamazepine  Lithium  Valproate |

**Supplementary Table 2:** **Sedative drugs used to determine sleep disorder status**

| **Drugs** | **Dosages** | **Conditions** |
| --- | --- | --- |
| **Antidepressants** | | |
| Agomelatine | 25-50mg/day |  |
| Mianserin | 30-90mg/day | Indication confirmed in medical files or discharge letter. |
| Mirtazapine | 15mg/day | Indication confirmed in medical files or discharge letter. |
| Trazodone | 25-100mg/day | Indication confirmed in medical files or discharge letter.  If over 65 years old, indication confirmed in the clinical report¹ |
| **Antipsychotics** | | |
| Clotiapine | 20-40mg/day | Indication confirmed in medical files or discharge letter. |
| Levomepromazine | 25-50mg/day | Indication confirmed in medical files or discharge letter. |
| Quetiapine | 25-50mg/day | Indication confirmed in medical files or discharge letter except for 41 patients which were confirmed by quetiapine low dosage after 8PM.²  If over 65 years old, indication confirmed in the clinical report¹ |
| **Benzodiazepines** | | |
| Alprazolam |  | Indication confirmed in medical files or discharge letter. |
| Bromazepam |  | Indication confirmed in medical files or discharge letter. |
| Clorazepate |  | Indication confirmed in medical files or discharge letter. |
| Diazepam |  | Indication confirmed in medical files or discharge letter. |
| Flunitrazepam |  | Indication confirmed in medical files or discharge letter. |
| Flurazepam | 15-30mg/day | Indication confirmed in the medical files |
| Lorazepam |  | Indication confirmed in medical files or discharge letter. |
| Midazolam | 15-30mg/day | Indication confirmed in medical files or discharge letter. |
| Oxazepam | 15-30mg/day | Indication confirmed in medical files or discharge letter. |
| Triazolam |  | Indication confirmed in medical files or discharge letter. |
| **Herbal sedatives³** | | |
| Hops |  |  |
| Valerian |  |  |
| **Hypnotic, Z-drugs³** | | |
| Zolpidem |  |  |
| Zopiclone |  |  |
| **Other drugs** | | |
| Clomethiazole |  | Only if administered after 8PM  or indication confirmed medical files |
| Chloral Hydrate | 500-1000mg/day | Only if administered after 8PM  or indication confirmed in medical files |
| Diphenhydramine | 25-50mg/day | Only if administered after 8PM  or indication confirmed in medical files |
| Doxylamine | 10-50mg/day | Only if administered after 8PM  or indication confirmed in medical files |
| Hydroxyzine | 25-50mg/day | Only if administered after 8PM  or indication confirmed in medical files |
| Melatonin³ |  |  |

¹ In elderly patients trazodone and quetiapine can be prescribed at low doses for other medical conditions, they were all confirmed by discharge letter or prescription condition in medical files.

² 41 inpatients were considered as having sleep disorders based only on quetiapine low dosage.

³ Medication used to define sleep disorders in outpatients

- **SKIPOGH1 (recruitment between November 2009 and April 2013)**

1129 individuals

Excluding individuals without plasma methylxanthine levels*

**1123** individuels

- **SKIPOGH2 (recruitment between March 2013 and December 2016)**

1034 individuals

Excluding individuals without plasma methylxanthine levels*

**926** individuels

**Supplementary Figure 1: Procedure for participants’ inclusion from SKIPOGH study**

* Levels of caffeine, paraxanthine, theophylline and theobromine were quantified by ultra-high-performance liquid chromatography coupled to a tandem quadrupole mass spectrometer with electrospray ionization. The limit of quantification for all analytes was 5 ng/mL.

**
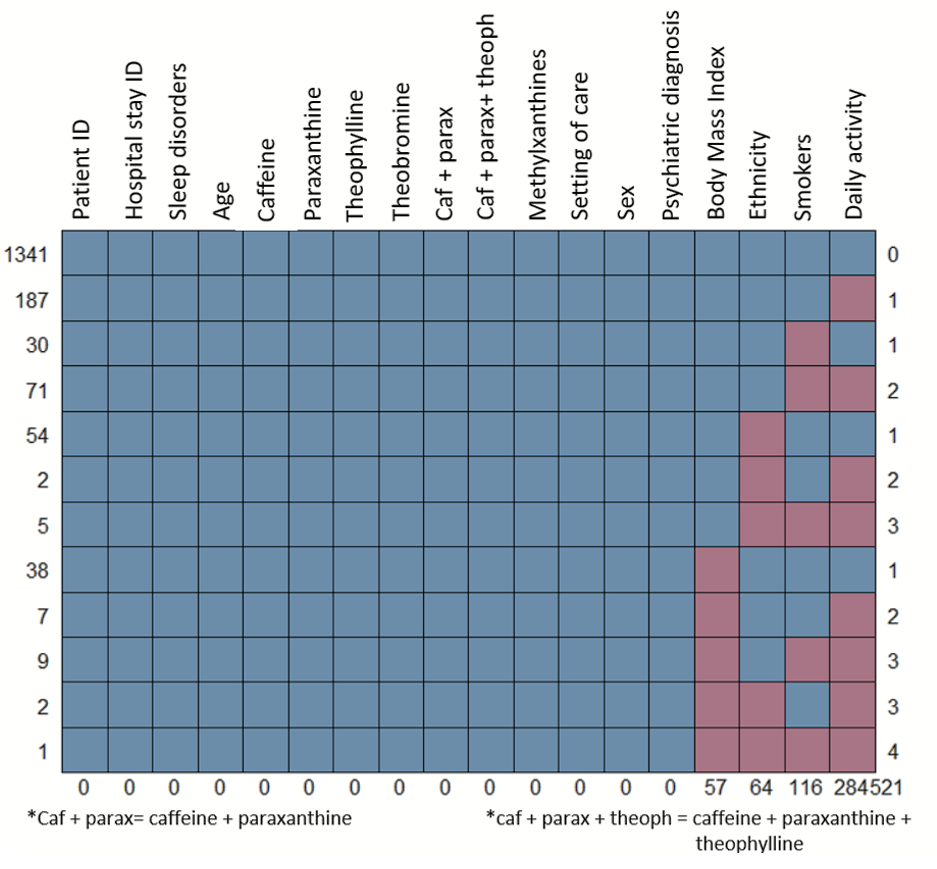
**

**Supplementary Figure 2: Number of missing data by variable and observations**

13919 observations

(3678 patients)

Excluding patients not from PsyMetab and observations without blood samples

2975 observations

(1589 patients)

Excluding observations with only plasma methylxanthine levels and no clinical data (age and gender, BMI, smoking, psychiatric diagnosis, psychotropic medication, etc.)

2442 observations

(1288 patients)

Excluding outpatients taking sedative medication(s)* except melatonin, zolpidem, zopiclone and herbal sedatives

**1747** observations

(**1060** patients)

included in clinical analyses

Excluding non-Caucasians

Only the first observation per patient was kept for statistical analysis

**669** observations/patients

included in PRS analyses

**Supplementary Figure 3: Procedure for participants’ inclusion in the study**

*Because neither the doses nor the timing of the intake of sedative medication(s) were available for outpatients, sleep disorders could not be ascertained and such patients were therefore excluded.

**Supplementary Table 3: Clinical and demographic data comparing outpatients and inpatients**

|  | Outpatients  (N=834) | Inpatients  (N=913) | p-Value |
| --- | --- | --- | --- |
| Age (median, IQR) years | **40 (28-51)** | **46 (29-61)** | **<10^-4^** |
| Sex (Female) (N, %) | **385 (46)** | **486 (53)** | **0.003** |
| BMI (median, IQR) kg/m² | **26.4 (22.4-29.5)** | **25 (21.2-27.7)** | **<10^-4^** |
| Smokers (N, %)  Yes  No | **422 (53)**  372 (47) | **396 (47)**  441 (53) | **0.02** |
| Psychiatric diagnosis (N, %) ^a^  Other disorders  Psychotic disorder  Schizoaffective disorder  Bipolar disorder  Depression | 148 (18)  267 (32)  118 (14)  128 (15)  173 (21) | 149 (16)  344 (38)  122 (13)  132 (15)  166 (18) | 0.17 |
| Daily activity (N, %)  <30 min/day  30 – 60 min/day  >60min/day | 231 (30)  265 (35)  **270 (35)** | 389 (56)  205 (29)  **103 (15)** | **<10^-3^** |
| Blood pressure (median, IQR) mmHg  Systolic  Diastolic | 123 (111-132)  80 (73-89) | 122 (110-133)  77 (69-84) | 0.79  **<10^-4^** |
| Creatinine (median, IQR) µmol/l | 76.7 (67-86) | 78.2 (66-86) | 0.90 |

Bold values indicate significant results

Y: Years; IQR: Interquartile range; N: Number; BMI: Body Mass Index; kg: Kilogram; m²: Square meter; min: Minutes; mmHg: Millimeter of mercury; µmol: Micromole; l: Liter

ᵃPsychiatric diagnoses were based on ICD-10 classification, and were classified as: **Other disorders** [F00-F19; F34-F99] | **Psychotic disorders** [F20-F24; F28-F29] | **Schizoaffective disorders** [F25] | **Bipolar disorders** [F30-F31] | **Depression** [F32-F33]

N varies between variables due to missing values (see Methods)


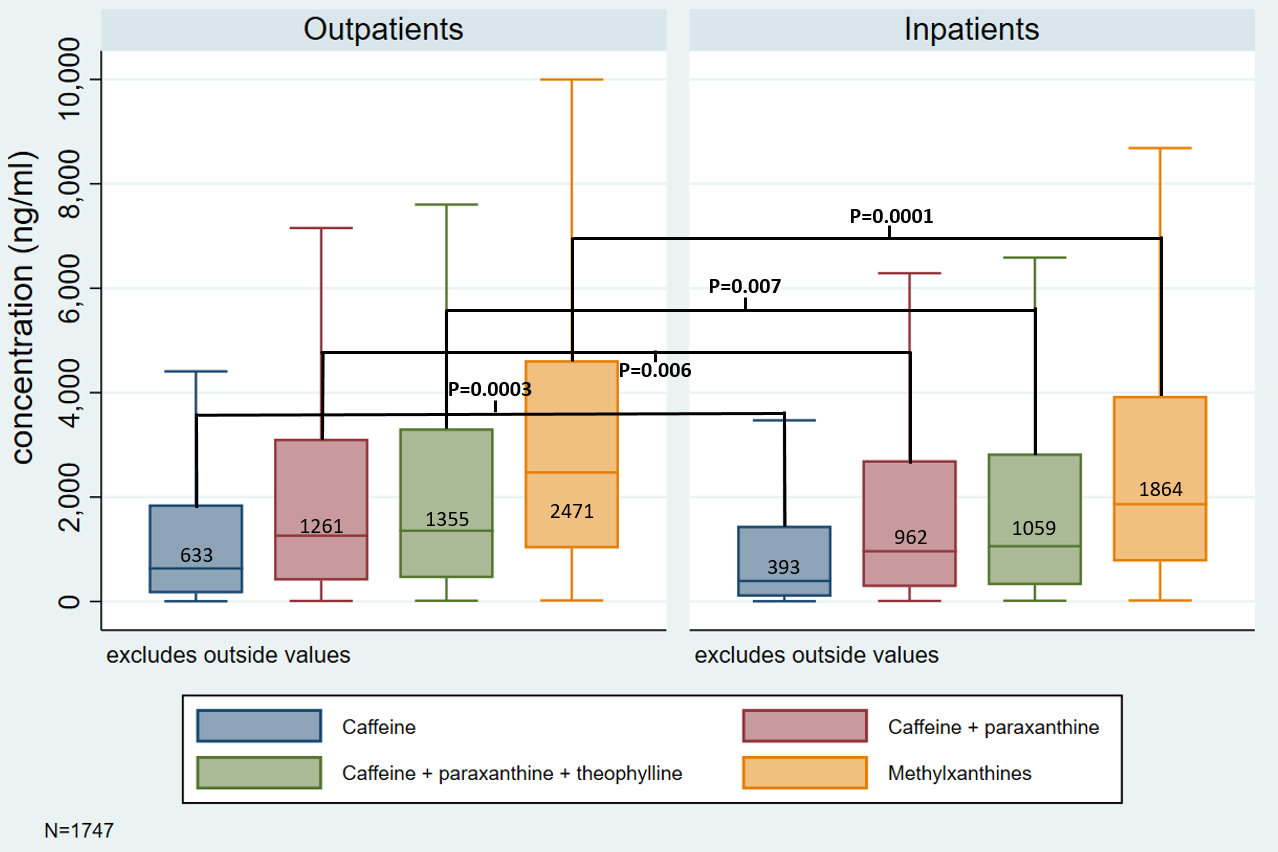


**Supplementary Figure 4:** **Plasma methylxanthine levels in outpatients and inpatients**

ng: nanogram; ml: milliliter, N: number; Methylxanthines: caffeine + paraxanthine + theophylline + theobromine.

Bold values indicate significant results

**Supplementary Table 4:** **Association between sleep disorders, clinical variables and quantiles of plasma levels of caffeine + paraxanthine + theophylline in inpatients (N=913)**

|  | OR | 95% CI | P.value |
| --- | --- | --- | --- |
| Age (10 Years) | **1.27** | 1.15 – 1.41 | **<10^-3^** |
| Sex (Female) | 1.12 | 0.8 – 1.55 | 0.51 |
| BMI (kg/m²) | **1.04** | 1.01 – 1.08 | **0.01** |
| Smokers (Yes) | 1.25 | 0.88 – 1.79 | 0.21 |
| Q6 vs Q1 * | 2.14 | 0.98 – 4.65 | 0.056 |
| Q5 vs Q1 * | 1.19 | 0.64 – 2.23 | 0.58 |
| Q4 vs Q1 * | 1.08 | 0.62 – 1.9 | 0.78 |
| Q3 vs Q1 * | 1.33 | 0.76 – 2.31 | 0.31 |
| Q2 vs Q1 * | 1.28 | 0.7 – 2.32 | 0.42 |
| Psychotic disorders ** | **1.65** | 1.01 – 2.7 | **0.04** |
| Schizoaffective disorders ** | **2.81** | 1.48 – 5.33 | **0.001** |
| Bipolar disorders ** | **1.99** | 1.1 – 3.59 | **0.02** |
| Depression ** | **2.17** | 1.25 – 3.79 | **0.006** |
| Length of stay (≥62 days) *** | 0.91 | 0.65 – 1.28 | 0.60 |

Bold values indicate significant results. CI: Confidence Interval; BMI: Body Mass Index; kg: Kilogram; m²: Square meter; Log: Logarithm

* Log (caffeine + paraxanthine + theophylline; in ng/ml): Q1≤4.5; 4.5<Q2≤5.75; 5.75<Q3≤6.96; 6.96<Q4≤7.94; 7.94<Q5≤8.64; Q6>8.64

**Psychiatric diagnoses were based on ICD-10 classification and were classified as: Other disorders [F00-F19; F34-F99] | Psychotic disorders [F20-F24; F28-F29] | Schizoaffective disorders [F25] | Bipolar disorders [F30-F31] | Depression [F32-F33]

**Diagnoses were compared to “other disorders”

***62 days is the median length of stay

Similar results were found after excluding the 41 patients considered to have sleep disorders on the basis of low-dose quetiapine alone (data not shown).

**Supplementary Table 5:** **Association between sleep disorders, clinical variables and quantiles of plasma levels of caffeine in inpatients (N=913)**

|  | OR | 95% CI | P.value |
| --- | --- | --- | --- |
| Age (10 Years) | **1.27** | 1.14 – 1.40 | **<10^-3^** |
| Sex (Female) | 1.10 | 0.79 – 1.53 | 0.57 |
| BMI (kg/m²) | **1.04** | 1.01 – 1.08 | **0.01** |
| Smokers (Yes) | 1.26 | 0.88 – 1.81 | 0.21 |
| Q6 vs Q1 * | **3.12** | **1.34 – 7.26** | **0.008** |
| Q5 vs Q1 * | 1.03 | 0.55 – 1.94 | 0.91 |
| Q4 vs Q1 * | 1.14 | 0.64 – 2.02 | 0.66 |
| Q3 vs Q1 * | 1.45 | 0.83 – 1.89 | 0.19 |
| Q2 vs Q1 * | 1.04 | 0.58 – 1.89 | 0.89 |
| Psychotic disorders ** | **1.65** | 1.01 – 2.7 | **0.048** |
| Schizoaffective disorders ** | **2.78** | 1.46 – 5.28 | **0.002** |
| Bipolar disorders ** | **2.01** | 1.11 – 3.63 | **0.02** |
| Depression ** | **2.1** | 1.2 – 3.67 | **0.009** |
| Length of stay (≥62 days) *** | 0.92 | 0.66 – 1.29 | 0.61 |

Bold values indicate significant results. CI: Confidence Interval; BMI: Body Mass Index; kg: Kilogram; m²: Square meter; Log: Logarithm

* Log (caffeine; in ng/ml): Q1≤3.17; 3.17<Q2≤4.52; 4.52<Q3≤5.97; 5.97<Q4≤7.27; 7.27<Q5≤8.14; Q6>8.14

**Psychiatric diagnoses were based on ICD-10 classification and were classified as: Other disorders [F00-F19; F34-F99] | Psychotic disorders [F20-F24; F28-F29] | Schizoaffective disorders [F25] | Bipolar disorders [F30-F31] | Depression [F32-F33]

**Diagnoses were compared to “other disorders”

***62 days is the median length of stay

Similar results were found after excluding the 41 patients considered to have sleep disorders on the basis of low-dose quetiapine alone (data not shown).

**Supplementary Table 6:** **Association between length of stay* and plasma methylxanthine levels (N=650)**

|  | Estimates | 95% CI | p-value |
| --- | --- | --- | --- |
| Log (caffeine + paraxanthine + theophylline) | -0.02 | -0.06 – 0.02 | 0.39 |
| Age (10 Years) | **0.19** | 0.15 – 0.23 | **<10^-3^** |
| Sex (Female) | **-0.19** | -0.33 – -0.06 | **0.006** |
| BMI (kg/m²) | -0.01 | -0.02 – 0.00 | 0.12 |
| Smokers (Yes) | -0.14 | -0.29 – 0.00 | **0.05** |
| Psychotic disorders ** | **0.38** | 0.15 – 0.61 | **0.001** |
| Schizoaffective disorders ** | 0.26 | -0.00 – 0.53 | **0.05** |
| Bipolar disorders ** | **0.27** | 0.01 – 0.52 | **0.04** |
| Depression ** | 0.14 | -0.09 – 0.39 | 0.22 |
| Sleep disorders | -0.06 | -0.22 – 0.09 | 0.42 |

Bold values indicate significant results. CI: confidence interval; BMI: Body Mass Index; kg: Kilogram; m²: Square meter; Log: Logarithm

*In order to reduce variability, length of stay was transformed to logarithm scale. First observation per patient was kept

**Psychiatric diagnoses were based on ICD-10 classification and were classified as: Other disorders [F00-F19; F34-F99] | Psychotic disorders [F20-F24; F28-F29] | Schizoaffective disorders [F25] | Bipolar disorders [F30-F31] | Depression [F32-F33]

**Diagnoses were compared to “other disorders”

**Supplementary Table 7: Association between sleep disorders and plasma caffeine + paraxanthine + theophylline levels in outpatients (N=834)**

|  | OR | 95% CI | P-value |
| --- | --- | --- | --- |
| Age (10 Years) | 1.09 | 0.97 – 1.22 | 0.14 |
| Sex (Female) | 0.97 | 0.69 – 1.35 | 0.85 |
| BMI (kg/m²) | 1.01 | 0.98 – 1.04 | 0.48 |
| Smokers (Yes) | 0.94 | 0.66 – 1.34 | 0.74 |
| Log (caffeine + paraxanthine+ theophylline): Q6* vs all other patients | **1.66** | 1.17 – 2.36 | **0.005** |
| Psychotic disorders ** | 0.88 | 0.55 – 1.4 | 0.58 |
| Schizoaffective disorders ** | **0.35** | 0.18 – 1.69 | **0.002** |
| Bipolar disorders ** | 0.81 | 0.47 – 1.4 | 0.45 |
| Depression ** | **0.5** | 0.32 – 0.93 | **0.02** |

Bold values indicate significant results. CI: confidence interval; BMI: Body Mass Index; kg: Kilogram; m²: Square meter; Log: Logarithm

*Log (Caffeine + paraxanthine + theophylline) (ng/ml): Q6>7.8

**Psychiatric diagnoses were based on ICD-10 classification and were classified as: Other disorders [F00-F19; F34-F99] | Psychotic disorders [F20-F24; F28-F29] | Schizoaffective disorders [F25] | Bipolar disorders [F30-F31] | Depression [F32-F33]

**Diagnoses were compared to “other disorders”

**Supplementary Table 8: Plasma methylxanthine levels in outpatients from PsyMetab and SKIPOGH1 participants:**

| Median, IQR | SKIPOGH1 (N=1123) | PsyMetab (N=550) | p-Value |
| --- | --- | --- | --- |
| Caffeine ng/ml | **591 (221-1323)** | **737 (175-1910)** | **0.01** |
| Caffeine + Paraxanthine ng/ml | 1276 (566-2453) | 1386 (467-3144) | 0.09 |
| Caffeine + paraxanthine + theophylline ng/ml | 1407 (632-2697) | 1477 (512-3356) | 0.18 |
| Methylxanthines ng/ml | **3087 (1608-5033)** | **2685 (1117-4760)** | **0.0037** |

Bold values indicate significant results

IQR: Interquartile range; ng: nanogram; ml: milliliter; Methylxanthines: caffeine + paraxanthine + theophylline + theobromine.

Wilcoxon-Mann-Whitney rank-sum tests was applied using the mean plasma methylxanthine levels per PsyMetab individual.

No significant differences of methylxanthine plasma levels were observed between inpatients of PsyMetab and SKIPOGH1 participants (data not shown)

**Supplementary Table 9: Plasma methylxanthine levels in outpatients from PsyMetab and SKIPOGH2 participants:**

| Median, IQR | SKIPOGH (N=926) | PsyMetab (N=550) | p-Value |
| --- | --- | --- | --- |
| Caffeine ng/ml | **504 (199-1165)** | **737 (175-1910)** | **0.0002** |
| Caffeine + Paraxanthine ng/ml | **1136 (494-2325)** | **1386 (467-3144)** | **0.0045** |
| Caffeine + paraxanthine + theophylline ng/ml | **1255 (546-2533)** | **1477 (512-3356)** | **0.01** |
| Methylxanthines ng/ml | **2165 (1210-3704)** | **2685 (1117-4760)** | **0.002** |

Bold values indicate significant results

IQR: Interquartile range; ng: nanogram; ml: milliliter; Methylxanthines: caffeine + paraxanthine + theophylline + theobromine.

Wilcoxon-Mann-Whitney rank-sum tests was applied using the mean plasma methylxanthines levels per PsyMetab individual.

No significant differences of methylxanthine plasma levels were observed between inpatients of PsyMetab and SKIPOGH2 participants (data not shown)

**Supplementary Table 10: Association between PRS of caffeine consumption, plasma caffeine and its metabolite levels and sleep disorders (N=669)**

| Variable | Beta - Coefficient | 95% CI | Corrected p-value* |
| --- | --- | --- | --- |
| Log (caffeine) | **0.10** | 0.03 - 0.17 | **0.01** |
| Log (paraxanthine) | **0.11** | 0.03 – 0.18 | **0.02** |
| Log (theophylline) | **0.09** | 0.02 – 0.16 | **0.02** |
| Log (theobromine) | -0.01 | -0.09 – 0.07 | 0.79 |
| Log (caffeine + paraxanthine + theophylline) | **0.10** | 0.03 – 0.18 | **0.01** |
| Sleep Disorders | -0.14 | -0.34 -0.05 | 0.18 |

Bold values indicate significant results. CI: confidence interval; Log: Logarithm

* Corrected by False Discovery Rate (6 tests)

**Supplementary Table 11: Association between PRS of sleep duration, plasma caffeine and its metabolite levels and sleep disorders (N=669)**

| Variable | Beta - Coefficient | 95% CI | Corrected p-value* |
| --- | --- | --- | --- |
| Log (caffeine) | 0.10 | -0.00 – 0.21 | 0.10 |
| Log (paraxanthine) | 0.13 | -0.01 – 0.27 | 0.09 |
| Log (theophylline) | 0.18 | 0.02 – 0.36 | 0.12 |
| Log (theobromine) | 0.09 | -0.06 – 0.25 | 0.26 |
| Log (caffeine + paraxanthine + theophylline) | 0.13 | -0.00 – 0.27 | 0.15 |
| Sleep Disorders | 0.02 | -0.4 – 0.5 | 0.90 |

CI: confidence interval; Log: Logarithm

* Corrected by False Discovery Rate (6 tests)
